# Antitumor Activity of a Polypyridyl Chelating Ligand: *In Vitro* and *In Vivo* Inhibition of Glioma

**DOI:** 10.1177/1759091415572365

**Published:** 2015-02-23

**Authors:** Clément N. David, Elma S. Frias, Catherine C. Elix, Kathryn E. McGovern, Ameae M. Walker, Jack F. Eichler, Emma H. Wilson

**Affiliations:** 1 Division of Biomedical Sciences, School of Medicine, University of California, Riverside, CA, USA; 2 Department of Chemistry, College of Natural and Agricultural Sciences, University of California, Riverside, CA, USA

**Keywords:** glioma, GL-26, polypyridyl chelating ligand, chemotherapy, mouse, tumor

## Abstract

Glioblastoma multiforme is an extremely aggressive and invasive form of central nervous system tumor commonly treated with the chemotherapeutic drug Temozolomide. Unfortunately, even with treatment, the median survival time is less than 12 months. 2,9-Di-*sec*-butyl-1,10-phenanthroline (SBP), a phenanthroline-based ligand originally developed to deliver gold-based anticancer drugs, has recently been shown to have significant antitumor activity in its own right. SBP is hypothesized to initiate tumor cell death via interaction with non-DNA targets, and considering most glioblastoma drugs kill tumors through DNA damage processes, SBP was tested as a potential novel drug candidate against glial-based tumors. *In vitro* studies demonstrated that SBP significantly inhibited the growth of rodent GL-26 and C6 glioma cells, as well as human U-87, and SW1088 glioblastomas/astrocytomas. Furthermore, using a syngeneic glioma model in mice, *in vivo* administration of SBP significantly reduced tumor volume and increased survival time. There was no significant toxicity toward nontumorigenic primary murine and human astrocytes *in vitro*, and limited toxicity was observed in *ex vivo* tissues obtained from noncancerous mice. Terminal deoxynucleotidyl transferase dUTP nick end labeling staining and recovery assays suggest that SBP induces apoptosis in gliomas. This exploratory study suggests SBP is effective in slowing the growth of tumorigenic cells in the brain while exhibiting limited toxicity to normal cells and tissues and should therefore be further investigated for its potential in glioblastoma treatment.

## Introduction

**Scheme 1.**
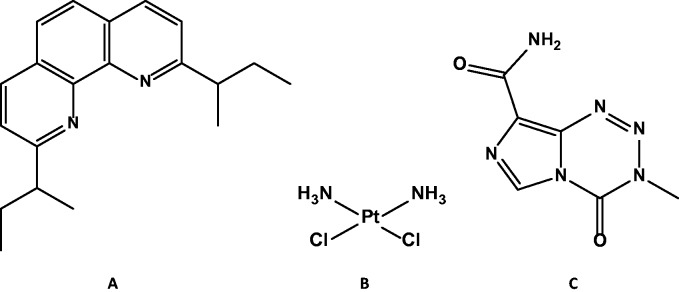
Molecular structure of **A**: 2,9-di-*sec*-butyl-1,10-phenanthroline (SBP); **B**: Cisplatin; and **C**: Temozolomide (TMZ).

Glioblastoma multiforme (GBM) is an extremely aggressive and invasive form of central nervous system (CNS) tumor with a survival prognosis of less than 1 year (Stupp et al., 2002; Hegi et al., 2004; Safdie et al., 2012; Poteet et al., 2013). Current therapies employ surgical removal in combination with radiation therapy and chemotherapy (Stupp et al., 2005). Though this removes a large part of the tumor, it often does not eliminate all tumor cells and relapses generally occur quickly. Furthermore, current chemotherapy and radiation therapy can leave patients with substantial deleterious side effects (Sughrue et al., 2009). Temozolomide (TMZ; see Scheme 1) is a chemotherapeutic drug that has been in use since 1999 to treat advanced glioblastomas and melanomas. Its antitumor effects stem from its capability to methylate DNA at the N-7 or O-6 position of guanine, thereby damaging the DNA and causing cell death (Srivastava et al., 1998; Hegi et al., 2004; Mutter and Stupp, 2006; Kim et al., 2010; Poteet et al., 2013). Unfortunately, virtually all patients relapse with TMZ-resistant disease, and many patients do not respond to TMZ (Hegi et al., 2005). The resistance to current chemotherapies, limited success of treatment, and poor long-term prognosis warrants the search for and creation of new drugs, which alone or in combination with other forms of therapy could target and eradicate tumor cells more efficiently (Hegi et al., 2004; Chamberlain et al., 2007).

Gold compounds have been long thought to possess strong antitumor activity, stemming from the fact that initial studies found some gold compounds were able to inhibit HeLa cell growth (Thang et al., 1976). Unfortunately, gold-based drugs were found to be unstable *in vivo* and had no therapeutic advantage over established chemotherapeutics (Thang et al., 1976; Wein et al., 2011). However, the subsequent development of coordinating ligands designed to stabilize gold complexes resulted in the discovery of the anticancer activities of gold(III) polypyridyl complexes, prompting a renewed interest in this area of drug design (Messori et al., 2000; Shi et al., 2006; Wein et al., 2011; Palanichamy et al., 2012). While the development of gold(III) drugs possessing polypyridyl ligand architectures has been progressing, some reports have indicated the polypyridyl ligands themselves exhibit antitumor activity similar to that of the parent gold complex, suggesting that the free ligand may play a role in the activity of this class of gold therapeutics. In a recent study of a gold(III) complex bearing the 2,9-di-*sec*-butyl-1,10-phenanthroline (SBP; see Scheme 1) polypyridyl ligand, control experiments found that the free SBP ligand exhibited remarkable *in vitro* activity against a variety of head-neck and lung (A549 and H1703 lung, and 886LN, Tu212, and Tu686 head/neck) cancer lines. In particular, the study revealed that SBP had *in vitro* IC_50_ values in the nanomolar concentration range, which were 20 to 100 times lower than the commonly used chemotherapy cisplatin (See Scheme 1) and 4 to 14 times lower than the parent gold(III) complex (Sanghvi et al., 2013). Thus, although metals complexed to phenanthroline-based ligands continue to be investigated for their antitumor properties (Narla et al., 2001; Scharwitz et al., 2008; Bieda, Ott, Gust, et al., 2009a; Bieda, Ott, Dobroschke, et al., 2009b; Dobroschke et al., 2009; Tan et al., 2010; Komor and Barton, 2013), our understanding of the properties and potential chemotherapeutic action of the ligands themselves remains an unexplored area of research.

One important finding in regard to the antitumor efficacy of SBP was that it had significant activity against the cisplatin-resistant H1703 lung tumor cell line, suggesting that SBP likely initiates tumor cell death via a mechanism involving a non-DNA target (Sanghvi et al., 2013). To date, the most successful drugs for treatment of glioblastoma, including TMZ, have been lipophilic alkylating agents that disrupt tumor cell growth through processes that initiate DNA damage (Ajaz et al., 2014). Cisplatin and other platinum-based drugs, which initiate tumor cell death by forming intrastrand crosslinks with DNA guanine base pairs, have also been found to demonstrate *in vitr*o activity against glioblastoma tumors (Wolff et al., 1999). Though platinum-based drugs have had more limited success *in vivo*, novel drug delivery approaches for glioblastoma treatment are being pursued (Charest et al., 2013; Miura et al., 2013). Given the aforementioned activity of SBP against cisplatin-resistant tumor cells, and the fact SBP is hypothesized to have non-DNA intracellular targets (Sanghvi et al., 2013), it was of interest to determine if this drug might have potential as a new lead compound for glioblastoma treatment. In particular, it was desired to determine whether a compound with a potentially different mechanism of antitumor activity might show promise as a therapeutic against glioblastoma tumors. Prior to carrying out detailed mechanistic studies on the antitumor activity of SBP, it was first desired to characterize the general *in vitro* efficacy of this drug against a panel of glioblastoma tumor cell lines and to determine if SBP has *in vivo* activity on an implanted murine glioma brain tumor model.

In the current study, a panel of rodent and human glioma cell lines was used for *in vitro* efficacy and toxicity assays. In addition, a syngeneic mouse model that recapitulates several aspects of human glioblastoma was used to investigate the antitumor capabilities of SBP *in vivo*. We report that SBP has significant *in vitro* activity against rodent (GL-26, C6) and human (U-87 and SW1088) glioblastoma/astrocytoma tumor cells and *in vivo* activity against implanted murine brain tumors. Finally, we provide preliminary studies on whether SBP limits tumor cell growth via cell cycle arrest or apoptosis.

## Materials and Methods

### Compound Synthesis

SBP was synthesized and purified according to previously reported protocols (Pallenberg et al., 1995; Jakobsen and Tilset, 2011).

### Cell Lines

The murine (C57BL/6) glioma cell line, GL-26, which is highly tumorigenic in C57BL/6 mice, was obtained as a generous gift from Dr. Pedro Lowenstein, University of Michigan, Ann Arbor (Candolfi et al., 2007; David et al., 2012). GL-26 cells were cultured in Dulbecco’s modified Eagle’s medium (DMEM)/F12 supplemented with 10% fetal calf serum (FCS), 1% penicillin/streptomycin, 1% L-glutamine, and 1% nonessential amino acids. Primary murine astrocytes were purified from C57BL/6 neonate brains and cultured in DMEM/F12 supplemented with 10% FCS, 1% nonessential amino acids, 1% L-glutamine, 50 IU/ml penicillin, 50 mg/ml streptomycin, and 10 mM Hepes buffer. U-87 (human glioblastoma), SW1088 (human astrocytoma), and C6 (rat glioma) were purchased from ATCC (cat# HTB-14, HTB-12 and CCL-107) and cultured following the ATCC’s guidelines. Primary human astrocytes were purchased from Sciencell (cat# HA-1800) and cultured following Sciencell’s recommendations. Human foreskin fibroblasts (HFFs) were cultured in DMEM/F12 supplemented with 10% FCS and 1% penicillin/streptomycin.

### Growth Assay

The sulforhodamine B (SRB) cytotoxicity assays were adapted from Skehan et al. (1990). Briefly, cells were plated at a density of 4,000 cells/well of a 96 well plate in a volume of 100 µL overnight at 37℃ and cultured in a humidified atmosphere of 5% CO_2_. Cells were exposed to SBP or TMZ at 0 to 25 µM for 48 hr before the culture supernatant was discarded, and the cells fixed for 1 hr with 10% cold trichloroacetic acid (100 µL per well). Cells used in recovery assay received fresh media for 48 hr following the 48 hr drug incubation before fixation. Fixed cells were then washed five times with de-ionized water, air dried, and stained with 0.4% SRB for 10 min (50 µL per well). After washing five times in 1% acetic acid and air-drying, bound SRB was dissolved in 10 mM unbuffered Tris base (pH 10.5; 100 µL per well). Bound SRB was then quantified by absorbance at 492 nm on a SpectraMax plate reader (Molecular Devices). The percent survival was then calculated based upon the absorbance values relative to control wells (0 µM SBP in 0.1% dimethyl sulfoxide). All cell growth assays were done such that each drug concentration was tested in triplicate, and each of these independent experiments was done three times.

### Propidium Iodide

GL-26 cells were plated at 4,000 cells/well in a 96 well plate in GL-26 media. The cells were treated 1 day postplating with 0 to 25 µM SBP for 48 hr. The cells were then detached with Trypsin/EDTA (Cellgro), washed and resuspended at 500,000 cells/ml in ice cold Na^+^/K^+^ balanced phosphate-buffered saline (PBS) and fixed by gently adding 70% ethanol and incubating for 2 hr at 4℃. GL-26 cells were then resuspended in 300 to 500 µL PI/Triton X-100 staining solution: 10 ml of 0. 1% (v/v) Triton X-100 (Sigma) in Na^+^/K^+^ balanced PBS with 2 mg DNAse-free RNAse A (Sigma) and 0.40 ml of 500 µg/ml PI (Roche). The stain was allowed to incubate at 37℃ for 15 min before data acquisition on a BD FacsCanto II flow cytometer. Independent experiments were carried out where only adherent cells were tested, as well as a combination of both adherent and detached cells were tested. Each of these experiments was repeated three times.

### *In Vivo* Experiments

All animal research was performed in accordance with the Animal Welfare Act. All protocols were approved by the Institutional Animal Care and Use Committee (IACUC) of the University of California, Riverside. Female C57BL/6 mice were obtained from Jackson Laboratories and maintained in a specific pathogen-free environment. Mice were anesthetized with continuous administration of 2.5% isofluorane. Cultured GL-26 cells were harvested by trypsinization, and 90,000 GL-26 cells in 3 µL of sterile Na^+^/K^+^ balanced PBS were injected intracranially. A stereotactic mouse frame was used to carry out the injection 1.0 mm anterior and 2.0 mm lateral to the junction of the coronal and sagittal sutures (bregma), and at a depth of 2.0 mm. Care was taken to alternate injection order and group assignment (treated vs. nontreated) to assure equal GL-26 cell viability between the two treatment groups. SBP was administered intravenously through the retro-orbital route at a concentration of 10 mg/kg in 200 µL sterile Na^+^/K^+^ balanced PBS. Drug was administered 1, 7, and 13 days after tumor implantation and sacrificed at Day 19 post implantation for tumor size analysis. A separate cohort was treated with 5 mg/kg SBP every 6 days and allowed to progress until moribund for a survival analysis (Untreated, *N* = 5; SBP-treated, *N* = 5).

### Histology

For brain tumor histology, mice were perfused intracardially with 4% formaldehyde in Na^+^/K^+^ balanced PBS, and brains were incubated in 4% formaldehyde overnight followed by 30% sucrose in Na^+^/K^+^ balanced PBS. Brains were flash frozen in isopentane, embedded in optimal cutting temperature (OCT) compound, coronally cryosectioned (12 µm), and stained with hematoxylin and eosin. Another cohort of equivalently drug-treated mice without tumors was used for liver, lung, and gut histology. In this instance, mice were sacrificed on Day 19, and the liver, lung, and gut tissues were collected and placed in 4% formaldehyde in Na^+^/K^+^ balanced PBS overnight. The organs were then placed for 48 hr in 70% EtOH before further dehydrating, paraffin embedding, and sectioning at 6 µm. Sections were then stained with hematoxylin and eosin, and pathology was assessed blindly and independently by a trained pathologist. A terminal deoxynucleotidyl transferase dUTP nick end labeling (TUNEL) staining kit was obtained from TREVIGEN (NeuroTACS II In Situ Apoptosis Detection Kit, Cat#4823-30-K) and used for both *ex vivo* slices and *in vitro* staining according to manufacturers instructions.

### Liver Toxicity

Intracardial blood was collected from the nontumor-bearing mice, allowed to clot, and then subjected to centrifugation for 10 min to collect serum. Aspartate transaminase (AST) and alanine transaminase (ALT) levels were measured in the serum using Bio Scientific (3913 Todd Lane Suite 312 Austin, TX) colorimetric kits (Cat#5605-01 and 3460-08, respectively).

### Statistical Analysis

All statistical analyses were done using GraphPad Prism software. Statistics on growth assays, tumor area, and AST/ALT concentrations were done using an unpaired two-tailed Student’s *t* test. A best-fit line was applied to the weights of tumor-bearing treated and nontreated mice. If the line deviates from a slope of 0, it indicates a change in the mouse weight over the recorded time. The survival curve was analyzed using a Mantel–Cox and a Gehan–Breslow–Wilcoxon test using GraphPad Prism software.

## Results

### SBP Inhibits Glioma Cell Growth *In Vitro*

**Figure 1.**
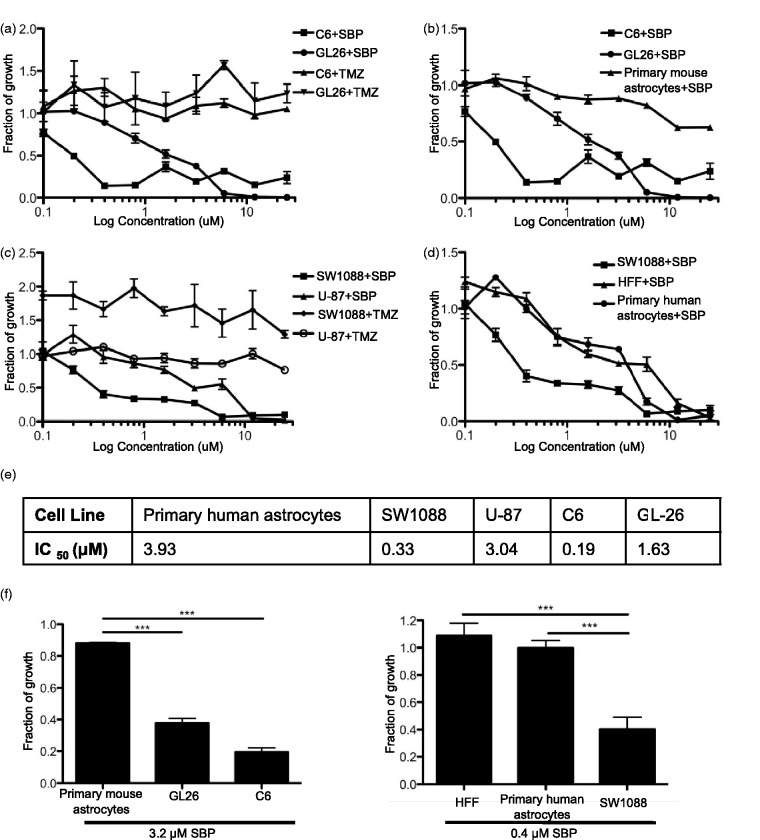
*In vitro* GL-26 inhibition with SBP: GL-26 and C6 cells were grown in a 96 well plate and treated with SBP or TMZ at 0.1 to 25 µM (a). To test SBP toxicity on nontumor cells, primary murine astrocytes were plated and treated as above (b). U-87 and SW1088 cells were grown in a 96 well plate and treated with SBP or TMZ at 0.1 to 25 µM (c). To test SBP toxicity on nontumor cells, primary human astrocytes and human foreskin fibroblasts (HFF) were plated and treated as above (d). IC_50_ (µM) displayed for each cell line tested (e). The largest toxicity window between normal and glioma cells plotted for both murine and human cells. (Student’s *t* test, primary murine astrocytes vs. GL-26 and C6 *p* < .0001; primary human astrocytes and HFF vs. SW1088 *p = *.0006 and *p = *.0007 respectively) (f). The sulforhodamine B (SRB) colorimetric assay was used to measure and plot fraction of growth of treated wells compared with nontreated controls. All cell growth assays were done in a minimum of triplicates and repeated a minimum of nine times. SBP = 2,9-Di-*sec*-butyl-1,10-phenanthroline; TMZ = Temozolomide.

To determine whether SBP had the capacity to inhibit the growth of glioma cell lines, GL-26 and C6 cells were cultured *in vitro* and incubated with concentrations of SBP and TMZ from 0.1 µM to 25 µM for 48 hr. The drug was then removed, and the effect of SBP on cell growth was assessed. A dose-related decrease in cell growth between 0.8 µM and 6 µM was observed in cells treated with SBP, whereas TMZ did not affect cell viability at any tested concentrations. At the IC_50_ value observed for SBP (1.63 µM) (Figure 1(a) and (e)), GL-26 growth was significantly reduced when compared with GL-26 treated with TMZ (no IC_50_ observed; *p = *.03) (Figure 1(a)). Similarly, at the IC_50_ for SBP (0.19 µM) (Figure 1(e)), C6 cell growth was significantly lower than in C6 cells treated with TMZ (no IC_50_ observed; *p = *.0112) (Figure 1(a)).

In order to assess the toxicity of the drug on normal cells, murine primary astrocytes were treated with SBP (0.1–25 µM) for 48 hr, and toxicity was quantified using an SRB assay. At the observed SBP IC_50_ of GL-26 cells (1.63 µM), primary murine astrocyte growth was inhibited by only 14% and no cell growth inhibition was observed at the IC_50_ of C6 cells (0.19 µM) treated with SBP (Figure 1(b)). This suggests that SBP has a therapeutic window of between 0.19 µM and 1.63 µM for the murine cells tested.

In an effort to assess the translational efficacy of SBP, the drug was tested against two human glioma lines. U-87 (human glioblastoma) and SW1088 (human astrocytoma) cells were incubated with the same concentration regime of SBP described above. At the IC_50_ of U-87 treated with SBP (3.04 µM) (Figure 1(e)), glioma growth is significantly reduced when compared with U-87 treated with TMZ (no IC_50_ observed; *p = *.0102) (Figure 1(c)). At the IC_50_ of SW1088 cells treated with SBP (0.33 µM) (Figure 1(e)), cell growth is significantly lower than in SW1088 cells treated with TMZ (no IC_50_ observed; *p = *.0005) (Figure 1(c)). To determine whether there was any toxicity of SBP on noncancerous human cells, primary human astrocytes and HFFs were treated with SBP. At the IC_50_ of SW1088 cells (0.33 μM) (Figure 1(e)), HFF cell growth and primary human astrocyte growth were unaffected (Figure 1(d)). This suggests the therapeutic window for SBP in human cells is between 0.33 µM and 3.04 µM.

In total, these results suggest SBP is significantly more effective at inhibiting the growth of these specific mouse and human tumor cells than the currently used glioma chemotherapy, TMZ. The concentration of SBP with the greatest efficacy against tumor cells and least toxicity in normal astrocytes was 3.2 µM for rodent cells and 0.4 µM for human cells (Figure 1(f)) *in vitro*.

### SBP Induces GL-26 Apoptosis

**Figure 2.**
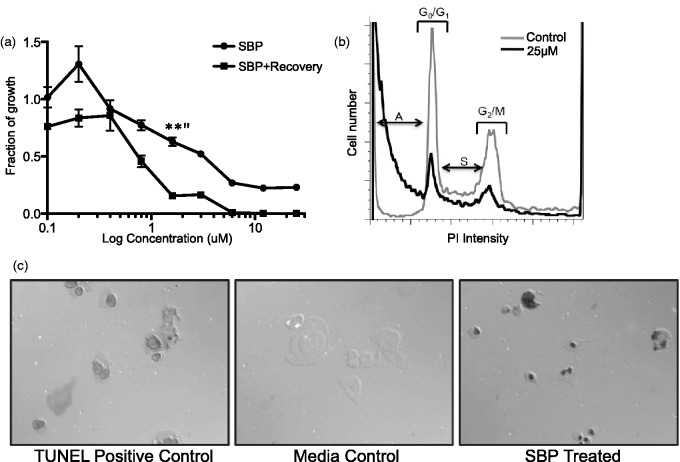
The compound SBP induces apoptosis: GL-26 cells were grown in a 96 well plate and treated with 0.4 to 25 µM SBP. After 48 hr incubation, the drug was removed and cells cultured for an additional 48 hr in fresh media. SRB was used to measure and plot fraction of growth of treated wells compared with a nontreated control. Nonrecovered GL-26 cells are plotted for reference (a). Propidium iodide staining intensity was measured by flow cytometry and plotted versus cell number to identify cell cycle stages (S: Synthesis; M: Mitotic) (b). SBP-treated and untreated cultured GL-26 cells were stained for apoptosis (TUNEL). The positive control was treated with the kit’s nuclease to generate DNA breaks in every cell (c). Both recovery and propidium iodide experiments were independently repeated a minimum of three times. SBP = 2,9-Di-*sec*-butyl-1,10-phenanthroline; SRB = sulforhodamine B; TUNEL = terminal deoxynucleotidyl transferase dUTP nick end labeling.

An important consideration is whether chemotherapeutics kill off and remove cancer cells or simply inhibit their growth. If cell growth is inhibited by the drug, continuous treatment is required to prevent regrowth. Therefore, preliminary studies were carried out in an effort to determine whether SBP induces tumor cell death or whether the drug simply disrupts cell growth. First, to test whether constant SBP administration is necessary to maintain growth inhibition, an SRB recovery assay was performed. Allowing the glioma cells to recover for 48 hr in fresh medium did not rescue cell growth. Instead significantly greater cell death was observed in the *recovered* versus acutely treated cells (*p = *.002) (Figure 2(a)). These data demonstrate that GL-26 cells continue dying after the removal of SBP and suggested that SBP targeted and disrupted cell survival rather than cell proliferation mechanisms.

To assess the effects of SBP on cell proliferation versus apoptosis, cell cycle analysis was performed using propidium iodide. Figure 2(b) demonstrates that SBP-treated cells were still capable of advancing to the S, G_2_, and mitotic phases and were thus not arrested in the G_1_ phase. However, the proportion of cells in these phases was reduced and treated cells had a significantly larger population of dead cells compared with untreated controls. To confirm whether cell death was the result of apoptosis, cultured GL-26 cells treated with SBP were subjected to TUNEL staining (Figure 2(c)). No positive staining was detected in the untreated sample, suggesting that no apoptosis occurred during the culturing of GL-26 cells in media alone (Figure 2(c)). The nuclease-treated positive control was uniformly TUNEL positive, and the dark staining in the SBP-treated GL-26 cells indicates that these cells are also undergoing apoptosis. These results demonstrate that SBP does not affect cell cycle progression, but rather kills GL-26 cells by inducing apoptosis.

### SBP Inhibits *In Vivo* Glioma Growth

Although no mouse model accurately mimics the generation of human GBM, intracranial injection of the GL-26 cell line leads to a morphologically similar and syngeneic tumor. It is also highly aggressive and invasive making it a valuable test for potential therapeutics. The tumors are extremely proliferative, and mice routinely die within 30 days following intracranial injection. This type of tumor implantation has been shown to lead to a robust tumor within a week (Candolfi et al., 2007; David et al., 2012; Safdie et al., 2012). After implanting the tumors, treatment mice were treated intravenously with SBP, and control mice were treated intravenously with saline solution on 1, 7, and 13 days post GL-26 cell injection.

**Figure 3.**
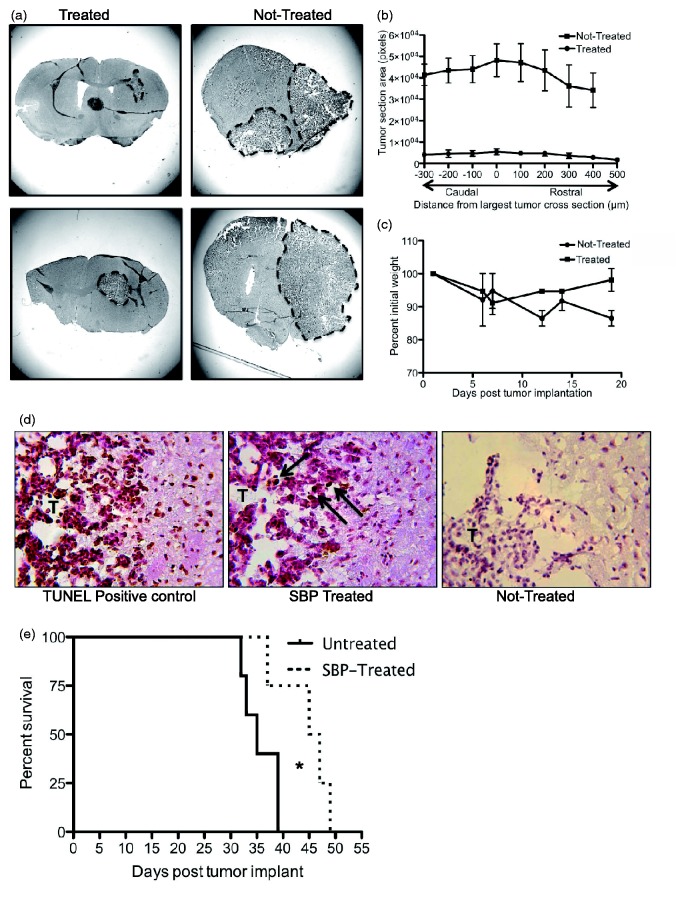
SBP inhibits *in vivo* glioma growth: Hematoxylin and eosin stained *ex vivo* coronal slices were taken from SBP-treated mice and nontreated mice (*N* = 3 for each group) (a) and tumor section areas quantified (Student’s *t* test, nontreated: 48187 ± 7736, SBP treated: 5489 ± 1369 *p* = .0056). T = tumor (b). Tumor-bearing mice were treated on Days 1, 7, and 13 post injection and sacrificed on Day 19. Mouse weights were recorded during the 19-day trial. A best-fit line (not shown) reveals a significant weight decrease in nontreated animals (*p* = .0261) but not in SBP-treated animals (*p* = .8792) (c). *Ex vivo* slices were stained for apoptosis (TUNEL) in SBP-treated (middle panel) and nontreated mice (right panel). Positive control was treated with the kit’s nuclease to generate DNA breaks in every cell (left panel) (d). For all experiments above, *N* = 4 for both treated and nontreated mice. GL-26 implanted mice were either left untreated (*N* = 5) or treated every 6 days with 10 mg/kg SBP (*N* = 5) and allowed to progress to moribund. A Mantel–Cox test *p* = .0384; and Gehan–Breslow–Wilcoxon test *p* = .0472 were used to test significance (e). SBP = 2,9-Di-*sec*-butyl-1,10-phenanthroline; TUNEL = terminal deoxynucleotidyl transferase dUTP nick end labeling.

All treatment and control mice were sacrificed at Day 19, and serial brain sections were stained with hematoxylin and eosin to reveal general morphology. In untreated mice, tumors were extensive. Glioma growth expanded from the striatum to most of the cortex of the injected hemisphere (Figure 3(a)). In contrast, mice treated with SBP had markedly smaller tumors (Figure 3(b)). Indeed, tumors in the SBP group were largely restricted to areas directly adjacent to the needle tract, and constrained to small areas of the striatum, although sometimes they expanded minimally to the cortex. In some cases, the untreated tumors expanded to the contralateral hemisphere, whereas the treated tumors never penetrated this region (Figure 3(a)). For each animal, tumor size was quantified by pixel area starting at the largest tumor cross-section (position 0) and measuring the tumor area in 100 µm intervals (rostral and caudal). Untreated animals were found to possess significantly larger tumors than the SBP-treated group (Not treated: 48187 ± 7736, SBP treated: 5489 ± 1369, *p* = .0056, measured at the largest tumor cross-section) (Figure 3(b)).

Body weights were recorded for 19 days after tumor implantation, and as expected, a decrease in weight was observed in both treated and nontreated groups during the first week post tumor implantation (Figure 3(c); Safdie et al., 2012). However, whereas mice treated with SBP regained nearly all their initial weight (>98%), mice left untreated exhibited continued weight loss (Figure 3(c)). A best-fit line (not shown) revealed that the nontreated group significantly deviated from zero (*p* = .0261), whereas the SBP-treated mice weights did not (*p* = .8792).

The *in vitro* assays demonstrated that SBP inhibited GL-26 cell growth via the induction of apoptosis. To determine whether apoptosis of GL-26 cells was a potential cause of the reduced tumor size *in vivo*, *in situ* TUNEL staining was conducted on serial brain sections from treated and untreated mice (Figure 3(d)). The untreated tumor displays faint positive TUNEL stain consistent with tumor growth and destruction of normal tissue (Kim et al., 2010). In contrast, SBP-treated tumors revealed dark brown cytoplasmic and nuclear staining indicative of cell necrosis and increased DNA fragmentation, respectively (Figure 3(d)). These data are consistent with SBP causing cell death via apoptosis as seen *in vitro*.

To test whether decreased tumor size and increased tumor cell apoptosis translates to an increase in survival time, mice were allowed to progress to moribund state and survival monitored. Mice treated with SBP survived significantly longer than untreated mice (Mantel–Cox test *p* = .0384; and Gehan–Breslow–Wilcoxon test *p* = .0472) with untreated mice living a median of 35 days and SBP-treated mice living a median of 46 days (Figure 3(e)). In combination with the *ex vivo* TUNEL, these data suggest that SBP treatment slows tumor growth, likely via apoptosis, and leads to improved survival.

### SBP Does not Cause Overt Peripheral Pathology

**Figure 4.**
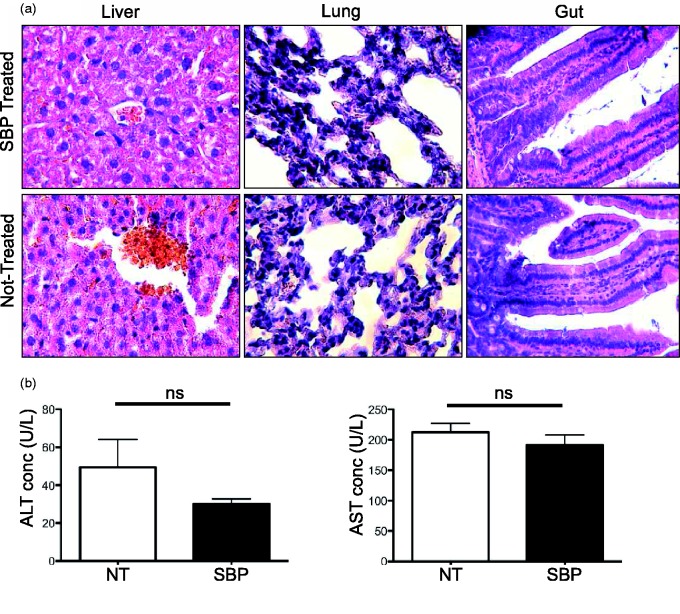
SBP does not cause peripheral pathology: Sections (6 µm), from liver, lung, and gut were obtained from SBP-treated and nontreated mice, stained with hematoxylin and eosin, and assessed blindly by a trained pathologist (a) (*N* = 4 for both treated and nontreated mice). Blood samples from treated and untreated mice were tested for levels of alanine aminotransferase and aspartate aminotransferase (Student’s *t* test, ALT: *p* = .2596 and AST *p* = .3982) (b) (*N* = 4 for both treated and nontreated mice). SBP = 2,9-Di-*sec*-butyl-1,10-phenanthroline.

Even though minimal inhibition of primary astrocytes was observed in both murine and human cells, *in vivo* administration can cause accumulation and breakdown products not observed *in vitro*. To determine the *in vivo* toxicity of SBP, nontumor-bearing mice were treated with either saline or 10 mg/kg SBP. After administering these treatments on Days 1, 7, and 13, the same regime for tumor inhibition, both the untreated control mice and SBP-treated mice were euthanized on Day 19. The liver, lungs, and proximal small intestines were subsequently collected for histopathological analysis. In the duodenum, no general pathology that might have resulted from inhibition of cell division was detected, as there were no differences in crypt or villus architecture, or goblet cell density between treated and untreated mice. Analysis of lung tissue also revealed no overt pathology. Treated liver sections revealed minor endothelial damage, but no overt hepatocyte damage (Figure 4(a)). Serum concentrations of liver enzymes were also measured to provide an indication of potential toxicity (Danan and Benichou, 1993; Amacher, 1998). Neither AST nor ALT concentrations, early indicators of toxicity caused by an intravenously administrated drug (Danan and Benichou, 1993; Scheig, 1996; Amacher, 1998), were significantly different between treated and nontreated mice (Figure 4(b)). These results reveal that SBP results in negligible *in vivo* toxicity when 10 mg/kg SBP is administered to mice over a 19-day period.

## Discussion

With current combination therapies extending the survival rate of patients with gliomas by a mean of 8 to 18 months (Chamberlain et al., 2007; Poteet et al., 2013), more potent compounds are desperately needed to control tumor growth. In this study, we used the GL-26 cell line as a model for glioblastoma. The GL-26 cells exhibit similar aggressive, proliferative, and tumorigenic properties as gliomas seen in humans and express the mouse version of CD133, associated with tumor stem cells (Candolfi et al., 2007; Golebiewska et al., 2013). Although implantation of GL-26 cells has limitations compared with a xenotransplant that enables *in vivo* testing on human tumor cells or a genetic model that enables the evaluation of cancer-initiating events (Chen et al., 2012), these cells, when injected into the mouse striatum establish a large and rapidly growing tumor that is morphologically similar to GBM (Stupp et al., 2002; Candolfi et al., 2007; Smith et al., 2009; Sughrue et al., 2009; David et al., 2012; Golebiewska et al., 2013). It also allows analysis in an immunologically intact animal where the tumor is tolerated in the murine brain. The resulting highly invasive tumor makes it a strong and therefore valuable test for potential therapies (Candolfi et al., 2007; Smith et al., 2009; Safdie et al., 2012).

Previously, SBP was found to have remarkable *in vitro* activity against a panel of three head-neck and two lung tumor lines, and when tested against a cisplatin-resistant tumor was found to have an IC_50_ value approximately 100 times lower than cisplatin (Sanghvi et al., 2013). The prime mechanism of cisplatin is thought to be intercalation of DNA (Komor and Barton, 2013). Such a significant reduction in IC_50_ by SBP could therefore point to a novel mechanism not dependent on DNA interactions. Glioma treatment is notoriously difficult, and the limitations are often due to tumor resistance to typical DNA targeting antiglioma drugs. Thus, we sought to test the *in vitro* anticancer activity of SBP against the rodent C6 and GL-26, and the human U-87 and SW1088 cell lines and compare it with the clinically used glioblastoma drug TMZ. Even though previous reports have shown the *in vitro* activity of polypyridyl ligands (Thang et al., 1976; Chamberlain et al., 2007; Roy et al., 2008; Wesselinova et al., 2009; Wein et al., 2011; Palanichamy et al., 2012), this report is to our knowledge the first study on the *in vitro* and *in vivo* anticancer activity of this class of compounds against glioblastoma tumors. The data reported here demonstrate strong *in vitro* toxicity of SBP against glioma cell lines at low micromolar concentrations, with significantly diminished toxicity to both noncancerous human fibroblasts and noncancerous primary human and murine astrocytes. In addition, we demonstrate that intravenously administered SBP can significantly reduce the growth of an intracranially implanted murine brain tumor resulting in an increase in survival similar to the TMZ murine glioma model (Kim et al., 2010).

A major concern in new drug development is the side effects associated with the drug. We have demonstrated high levels of cytotoxicity against GL-26, C6, and SW1088 at low micromolar concentrations, with minimal toxicity toward primary murine and human astrocytes and the noncancerous HFF cell line. AST and ALT are often used as markers for liver health during chemotherapy, where elevated levels indicate liver damage and the AST/ALT ratio can further be used to differentiate between the causes of liver damage (Danan and Benichou, 1993; Scheig, 1996; Amacher, 1998). Following treatment with SBP, AST, and ALT levels were similar to untreated mice and fell well within their respective normal physiological ranges. Furthermore, histopathalogical analysis of the proximal gut, lung, and liver only revealed minor damage to liver endothelial cells. These results mirror the low toxicity observed in TMZ-treated patients (Friedman et al., 2000; Su et al., 2004; Pouratian et al., 2007; Niewald et al., 2011). Perhaps more importantly, SBP treatment resulted in smaller, more contained tumors. This is especially relevant, as contained tumors are easier to remove surgically (Duffau, 2009). Thus, the route of administration, the strong antitumor properties, and the low toxicity to normal cells suggest SBP has potential for future anticancer development.

Even though *in vivo* experiments corroborated the *in vitro* data that demonstrated increased apoptosis in SBP-treated groups compared with nontreated controls, the obvious question remains: What is the mechanism by which SBP induces apoptosis? More specifically, is SBP-induced apoptosis a result of a similar mechanism as TMZ, which is thought to methylate guanine residues in the DNA (Srivastava et al., 1998; Hegi et al., 2004; Mutter and Stupp, 2006; Chamberlain et al., 2007; Kim et al., 2010; Poteet et al., 2013)? At first glance, it might be expected that SBP acts as a DNA intercalator, as the compound has significant aromatic character. This class of compounds is known to have significant interactions with DNA, which can result in disruption of DNA replication and induction of cell death in a similar fashion to the commonly used chemotherapy cisplatin (Kelland, 2006). However, as previously stated, SBP has enhanced antiproliferative effects on cisplatin-resistant cell lines. This suggests that the drug likely initiates tumor cell death via a mechanism not related to DNA interactions (Sanghvi et al., 2013). Additionally, the fact that TMZ exhibits no inhibition of tumor cell growth at any of the concentrations tested for SBP further corroborates the notion that SBP likely has a distinct DNA-independent mechanism. All of the tumor cell lines used in this study were desensitized to TMZ, a known DNA methylating agent, whereas all of the tumor cell lines were sensitive to SBP further suggesting that SBP acts differently than TMZ to inhibit growth. Finally, SBP has significantly reduced cytotoxicity toward noncancerous cells. Because DNA repair mechanisms are more vital to tumor cell proliferation compared with normal cell division, the observation that SBP has significantly stronger antiproliferative activity against tumor cell lines versus normal cells could point to SBP targeting enzymes involved in DNA repair. A report from Mendes et al*.* (2011) describes the poly(ADP-ribose) polymerase-1 (PARP-1) inhibition of gold(III) complexes possessing phenanthroline ligands that are in the same family as SBP. The gold(III) complex bearing the unsubstituted 1,10-phenanthroline was found in particular to be a potent PARP-1 inhibitor, involved in DNA repair mechanisms, and the authors attribute the gold complex’s activity to binding with the zinc finger motif in the enzyme. Given that SBP can act as a potent metal chelator, inhibition of DNA repair via binding to the zinc finger domain of PARP-1 is a plausible antitumor mechanism for this drug. Future research efforts in our lab will focus on testing this hypothesis.

In this report, the antitumor activity of SBP on glioblastomas was tested both *in vitro* and *in vivo.* The data demonstrate the potent antitumor activity of SBP *in vitro* with minimal toxicity to normal cells. The antitumor activity of SBP does not appear to be mediated by cell cycle disruption, but rather by inducing cell death as demonstrated by propidium iodide and TUNEL staining. SBP also reduced tumor size in an intracranial murine brain tumor without causing apparent pathology to normal tissues. Finally, SBP significantly increased survival time of mice intracranially injected with the GL-26 cell lines. Though further research should focus on the capability of SBP to stop or eradicate well-established tumors and the mechanism of action, the results described here clearly demonstrate that SBP has significant antiglioma activity, making it an important chemotherapy candidate for this aggressive, invasive, and difficult-to-treat class of tumor.
